# Sex differences and age of onset in well-being and recovery in people with psychotic disorders. A PHAMOUS study

**DOI:** 10.1192/j.eurpsy.2025.572

**Published:** 2025-08-26

**Authors:** L. Hoeksema, J. Bruins, S. Gangadin, L. van der Meer, M. Pijnenborg, E. Visser, M. Timmerman, S. Castelein

**Affiliations:** 1 Lentis Research, Lentis Psychiatric Institute; 2 Faculty of Behavioural and Social Sciences, Department of Clinical Psychology and Experimental Psychopathology, University of Groningen; 3 University Center for Psychiatry, Rob Giel Research center; 4 Department of Biomedical Sciences of Cells & Systems, University Medical Center Groningen; 5 Faculty of Behavioural and Social Sciences, Department of Clinical and Developmental Neuropsychology Psychopathology, University of Groningen; 6 Department of Psychiatric Rehabiliatation, Lentis Psychiatric Institute, Groningen; 7 Department of Psychotic Disorders, GGZ Drenthe Mental Health Institution, Assen; 8 University Medical Center Groningen, Faculty of Medical Sciences; 9 University Center for Psychiatry, Rob Giel Research center; 10 Faculty of Behavioural and Social Sciences, Department of Psychometrics & Statistics, University of Groningen, Groningen, Netherlands

## Abstract

**Introduction:**

Women generally have a later age of onset, and may therefore have a more favourable course of psychotic illness than men regarding psychopathology. Little is known about a broader range of outcomes, including well-being and recovery, and about the influence of age of onset.

**Objectives:**

This study examines longitudinal sex-related and age of onset-related differences in well-being and recovery of people with a psychotic disorder with long illness durations.

**Methods:**

Routine outcome monitoring data (2012-2021) of n=3843 patients were used. Well-being (quality of life and personal recovery) and recovery (clinical and societal recovery and psychosocial functioning) were assessed. Latent class growth analysis (LCGA) was performed to assess whether classes with different trajectories of well-being and recovery could be identified. Classes were related to sex and (early/late) age of onset of psychosis (EOP/LOP).

**Results:**

LCGA identified five classes with varying combinations in levels of well-being and recovery, which were stable over time. Sex, age of onset and the combination of these two were significantly related to class membership. Women and individuals with LOP were more prevalent in better functioning classes than men and individuals with EOP.

**Conclusions:**

This study showed sex differences in long-term recovery patterns of psychosis. Not only women but also individuals with LOP had a higher chance of better well-being and recovery, while men with EOP were at risk for worse outcomes. Taking these sex differences into account when deciding on policy and treatment protocols for individual patients might provide better mental health care to people with psychosis.

**Disclosure of Interest:**

None Declared

